# Development of an Allele-Specific PCR Assay for Simultaneous Sero-Typing of Avian Pathogenic *Escherichia coli* Predominant O1, O2, O18 and O78 Strains

**DOI:** 10.1371/journal.pone.0096904

**Published:** 2014-05-07

**Authors:** Shaohui Wang, Qingmei Meng, Jianjun Dai, Xiangan Han, Yue Han, Chan Ding, Haiwen Liu, Shengqing Yu

**Affiliations:** 1 Shanghai Veterinary Research Institute, Chinese Academy of Agricultural Sciences, Shanghai, China; 2 Key Lab of Animal Bacteriology, Ministry of Agriculture, Nanjing Agricultural University, Nanjing, Jiangsu, China; University of Malaya, Malaysia

## Abstract

Systemic infections by avian pathogenic *Escherichia coli* (APEC) are economically devastating to poultry industries worldwide. *E*. *coli* strains belonging to serotypes O1, O2, O18 and O78 are preferentially associated with avian colibacillosis. The *rfb* gene cluster controlling O antigen synthesis is usually various among different *E*. *coli* serotypes. In present study, the *rfb* gene clusters of *E*. *coli* serotypes O1, O2, O18 and O78 were characterized and compared. Based on the serotype-specific genes in *rfb* gene cluster, an allele-specific polymerase chain reaction (PCR) assay was developed. This PCR assay was highly specific and reliable for sero-typing of APEC O1, O2, O18 and O78 strains. The sensitivity of the assay was determined as 10 pg DNA or 10 colony forming units (CFUs) bacteria for serotypes O2 and O18 strains, and 500 pg DNA or 1,000 CFUs bacteria for serotypes O1 and O78 strains. Using this PCR system, APEC isolates and the infected tissue samples were categorized successfully. Furthermore, it was able to differentiate the serotypes for the samples with multi-agglutination in the traditional serum agglutination assay. Therefore, the allele-specific PCR is more simple, rapid and accurate assay for APEC diagnosis, epidemiologic study and vaccine development.

## Introduction

*Escherichia coli* typically colonize the mammalian and avian gastrointestinal tract and other mucosal surfaces. Although many *E. coli* strains are commensal, certain pathogenic strains can cause a wide variety of intestinal and extraintestinal diseases [1]–[2]. *E. coli* could be sero-typed by somatic (O), capsular (K), and flagellar (H) antigens [3], and a close connection exists among specific O-antigen serotypes and certain pathogenicity of pathogens. Avian pathogenic *E. coli* (APEC) are economically devastating to poultry industries worldwide. Previous studies indicated that varied serotypes including O1, O2, O18 and O78 are preferentially associated with APEC outbreaks, which accounted for more than 50% of the APEC issues [4]–[6]. Our previous epidemiology study showed that more than 85% APEC were O1, O2, O18 and O78 in the farms of Eastern China [7]–[8]. Moreover, there is less cross-reaction among serotypes. Thus, sero-typing of APEC bacteria isolated or in infected tissues would be a crucial modality for disease diagnosis, epidemiologic study and vaccine development.

APEC isolates are generally sero-typed by serum agglutination assay using specific O-antigen antiserum. This traditional assay needs isolated bacterial colony and specific antiserum for the sero-typing. Therefore, it is complex, costly, and time consuming. Moreover, cross-reactivity of the antisera with multiple O-antigen strains occurs occasionally. Recently, PCR-based method has been used as a rapid analytical technique for detection of a variety of bacterial strains [9]. Genes controlling O-antigen synthesis are in the *rfb* gene cluster, ranging from 4.2 to 20 kb, which is generally bordered by the *gnd* and *galF* genes in *E. coli*. Sequence analysis shows that the number and arrange of genes in the *rfb* gene cluster are various for different serotypes of *E. coli* [10]–[12]. Thus, PCR assays based on O-antigen gene clusters have been developed to determine predominant O serotypes of several pathogenic *E. coli* [13]–[21]. However, no rapid and sensitive PCR is available for sero-typing of APEC predominant O1, O2, O18 and O78 strains yet.

This study attempted to analyze the *rfb* gene clusters in APEC predominant serotypes O1, O2, O18 and O78 strains and develop an allele-specific PCR method for sero-typing of the O-antigens. The allele-specific PCR method was evaluated for its specificity, sensitivity, and application for APEC diagnosis.

## Materials and Methods

### Bacterial Strains, Growth Conditions and DNA Preparation

The bacterial strains used in this study are listed in Table 1. The *E. coli*, *Salmonella enterica* (*S. enterica*), *Riemerella anatipestifer* (*R. anatipestifer*) and *Pasteurella multocida* (*P. multocida*) strains were grown in appropriate medium at 37°C with aeration. Other reference strains from the Chinese Veterinary Culture Collection Center (CVCC, Beijing, China) or the American Type Culture Collection (ATCC, Manassas, VA, USA) were cultured in recommended conditions. Sixty-five APEC serotype O1, O2, O18 and O78 strains were isolated from chickens or ducks between 2010 and 2012, and kept in Shanghai Veterinary Research Institute, Chinese Academy of Agricultural Sciences (CAAS) [7]–[8]. Bacterial genomic DNA was prepared using TIANamp Bacterial DNA Kit (Tiangen, Beijing, China) according to the manufacturer’s guidelines.

**Table 1:** Bacterial strains used in this study.

Strains	Description	Source or reference
APEC O1	APEC strain, serotype O1	[22]
DE47	APEC strain, serotype O1	[7]–[8]
DE14	APEC strain, serotype O2	[7]–[8]
DE17	APEC strain, serotype O2	[7]–[8]
RS218	NMEC strain, serotype O18	[28]
CE66	APEC strain, serotype O18	[7]–[8]
DE48	APEC strain, serotype O78	[7]–[8]
DE65	APEC strain, serotype O78	[7]–[8]
CFT 073	UPEC strain, serotype O6	[29]
MG1655	*E. coli* strain, serotype O16	[30]
CVCC 1543	*E. coli* strain, serotype O38	CVCC a
CVCC 1547	APEC strain, serotype O73	CVCC
O131	*E. coli* strain, serotype O131	[31]
ATCC 43889	EHEC O157:H7	ATCC b
O138	*E. coli* strain, serotype O138	[31]
CVCC 3384	*S. enterica serovar typhimurium*	CVCC
CVCC 1805	*S. enterica serovar enteritidis*	CVCC
CVCC 519	*S. enterica serovar pullorum*	CVCC
CAU 0118	*S. enterica serovar anatum*	CVCC
CH3	*R. anatipestifer* strain, serotype 1	[27]
Th4	*R. anatipestifer* strain, serotype 2	[27]
HXb2	*R. anatipestifer* strain, serotype 10	[27]
CVCC 493	*Pasteurella multocida*	CVCC
CVCC 1651	*Mycoplasma gallisepticum*	CVCC
CVCC 274	*Mycoplasma avium*	CVCC
CVCC 543	*Staphylococcus aureus*	CVCC
IPDH 591-77	*Bordetella avium*	ATCC

a CVCC, Chinese Veterinary Culture Collection Center, China.

b ATCC, American Type Culture Collection, USA.

### Primer Design and Development of Allele-specific PCR Assay

**Figure 1:**
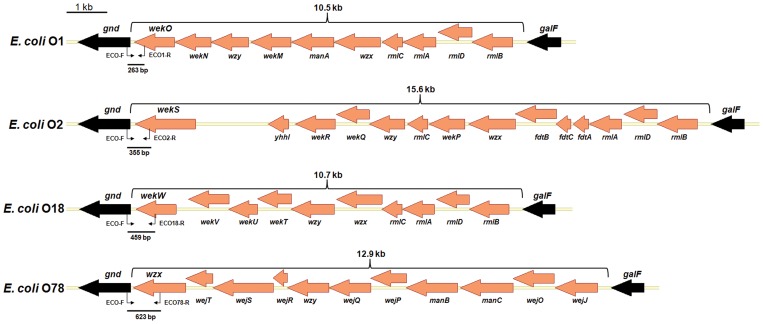
The *rfb* gene clusters of *E. coli* serotypes O1, O2, O18 and O78 strains. The black arrows correspond to *gnd* and *galF* genes. Grey arrows correspond to *rfb* gene cluster and the gene names are *italic* indicated. The length of *rfb* gene cluster was also shown. In the PCR reaction system, the universal forward primer was used for all the sero-typing amplification with specific reverse primers. The bold lines below the *gnd* gene indicate the size of the PCR products for different *E. coli* serotype strains, which allow the differentiation of the O types. Primers and their locations were also indicated.

The *rfb* gene cluster is generally bordered by the *gnd* and *galF* genes, which controls *E. coli* O-antigen synthesis and shows serotype-dependent differences in its gene sets and organization. To design the suitable primers, the *rfb* gene clusters of eight *E. coli* strains, including serotype O1 strains (strain APEC O1 [Acc No. CP000468.1] and G1632 [Acc No. GU299791.1]), serotype O2 strains (strain G1674 [Acc No. GU299792.1] and *E. coli* O2 [Acc No. EU549863.1]), serotype O18 strains (strains IHE3034 [Acc No. CP001969.1] and G1630 [Acc No. GU299793.1]), and serotype O78 strains (strain APEC O78 [Acc No. CP004009.1] and *E. coli* O78 [Acc No. FJ940775.1]) [21]–[26], were analyzed using software Vector NTI (Invitrogen, Carlsbad, CA, USA). The putative functions of the identified genes were determined based on their sequence similarity to genes of known function from the available databases, in which they were named according to the bacterial polysaccharide gene nomenclature (BPGN) system (www.microbio.usyd.edu.au/BPGD/default.htm). Then, the universal forward primer and specific reverse primers were designed based on the sequence of *gnd* gene and specific *rfb* genes to develop the allele-specific PCR assays (Table 2 and Fig. 1). Furthermore, the absence of amplification by our primers was tested *in silico* on the other available *rfb* gene cluster sequences.

**Table 2:** Primers used in this study.

Primer	Sequence (5′ to 3′)^a^	Target gene	Size of PCR product
ECO-F	CGATGTTGAGCGCAAGGTTG	*gnd*	
ECO1-R	CATTAGGTGTCTCTGGCACG	*rfbO1*	263 bp
ECO2-R	GATAAGGAATGCACATCGCC	*rfbO2*	355 bp
ECO18-R	AGAAGCATTGAGCTGTGGAC	*rfbO18*	459 bp
ECO78-R	TAGGTATTCCTGTTGCGGAG	*rfbO78*	623 bp

a The primers were designed based on the gene sequences of *wekO*, *wekS*, *wekW* and *wzx* in the *rfb* gene cluster of respective serotypes of *E. coli* strains.

The *E. coli* reference strains were used for the allele-specific PCR development. Briefly, 1 µL template DNA was added to the reaction mixture (25 µL) containing 2.5 µL 10× PCR buffer with MgCl_2_ (25 mM), 1.5 U *Taq* DNA polymerase (TaKaRa, Dalian, China), 2 µL dNTPs (2.5 mM for each dNTP), and 0.5 µL (10 µM) of each primer pair. The PCR reaction mixtures were subjected to the following conditions in ABI thermal cycler: pre-denaturation at 95°C for 5 min, followed by 30 cycles of 95°C for 35 s, 57°C for 30 s, 72°C for 40 s and a final extension at 72°C for 10 min. The PCR products were observed under ultraviolet light after electrophoresis on a 2% agarose gel.

### Specificity of the Allele-specific PCR

For the specificity analysis of the allele-specific PCR, *E. coli* reference strains, *S. enterica*, *R. anatipestifer*, and other species of bacteria strains were used as the templates (Table 1). The bacterial DNAs from *E. coli* serotypes O1, O2, O18 and O78 strains were used as positive controls. The negative control contained sterile distilled water in place of template DNA. Under optimized condition, each serotype-specific gene fragment was amplified with respective primer pairs. The PCR products were then cloned into pMD18-T vector (TaKaRa, Dalian, China) and DNA sequencing was performed on an Applied Biosystems DNA sequencer ABI PRISM 377. The reliability and specificity of the assay were also verified by comparing the sero-typing results of 65 APEC isolates to traditional serum agglutination assay. The traditional agglutination assay was carried out with rabbit anti-*E. coli* immune serum produced against *E. coli* O1, O2, O18 or O78 (Statens Serum Institut, Copenhagen, Denmark) according to the manufacturer’s guidelines. Briefly, the *E. coli* bacterial culture was boiled for 1 h. Then, the boiled culture was mixed with equal amount of O antiserum in glass tubes, which was incubated in a humid atmosphere at 50–52°C overnight. The reaction was read against artificial light with a black background. Physiological saline was used as a negative control and must be negative. If the negative control was positive, the strain was auto-agglutinating.

### Sensitivity of the Allele-specific PCR

The sensitivity was determined using diluted DNA templates, ranged from 100 ng to 1 pg. On the other hand, 10-fold serial dilutions of bacterial broth culture, ranged from 1×10^7^ to 1 colony forming units (CFUs), were also used as templates for the allele-specific PCR. The PCR assays were performed under the optimized condition.

### Sero-typing of APEC from Experimental and Clinical Infected Tissue Samples

APEC experimental infection was performed as described previously [27]. Briefly, 20 Cherry Valley ducks (7-day-old) were randomly divided into 5 groups. The ducks in groups 1–4 were infected intratracheally with a bacterial suspension containing APEC serotypes O1, O2, O18 or O78 strains at 10^8 ^CFUs, respectively. The ducks in group 5 were used as controls. At 24 h post-infection, ducks were dissected which livers were aseptically collected. The genomic DNAs were extracted and eluted with 100 µL of distilled water from each 100 mg of duck liver tissue using QIAamp DNA mini kit (Qiagen, Santa Clarita, CA, USA) according to the manufacturer’s instructions. The allele-specific PCR was carried out as described above. Genomic DNA of *E. coli* serotypes O1, O2, O18 or O78 strains were used as positive controls. In addition, 20 clinical diseased ducks with colibacillosis from APEC infected farms and 20 healthy ducks were obtained and subjected to APEC sero-typing using allele-specific PCR. To evaluate the results obtained from the PCR assays, all the samples were also subjected to an extended bacteriological examination and traditional serum agglutination assay.

### Ethics Statement

All procedures were carried out in accordance with guidelines of the Association for Assessment and Accreditation of Laboratory Animal Care International (AAALAC). The animal study protocol was approved by the Animal Care and Use Committee of Shanghai Veterinary Research Institute, CAAS, China.

## Results

### Bioinformatics Analysis of the APEC *rfb* Gene Clusters

*E. coli rfb* gene cluster mainly encodes glycosyltransferase, acetyltransferase, polysaccharide polymerase and flippase. Bioinformatics analysis showed that the gene numbers and arrange in the cluster were completely different among APEC serotypes. The cluster length between genes *gnd* and *galF* was 10.5 kb (10 ORFs), 15.6 kb (14 ORFs), 10.7 kb (10 ORFs), and 12.9 kb (11 ORFs) for serotypes O1, O2, O18 and O78 strains, respectively (Fig. 1). The putative functions of the identified genes were determined based on their sequence similarity to genes of known function from the available databases. As shown in Figure 1, the genes near to *gnd* in the cluster of serotype O1, O2, and O18 strains were glycosyltransferase encoding genes *wekO*, *wekS* and *wekW*, respectively. The gene near to *gnd* in the cluster of serotype O78 strains was O antigen flippase encoding gene *wzx*.

### Development of the Allele-specific PCR Assay for APEC Sero-typing

**Figure 2:**
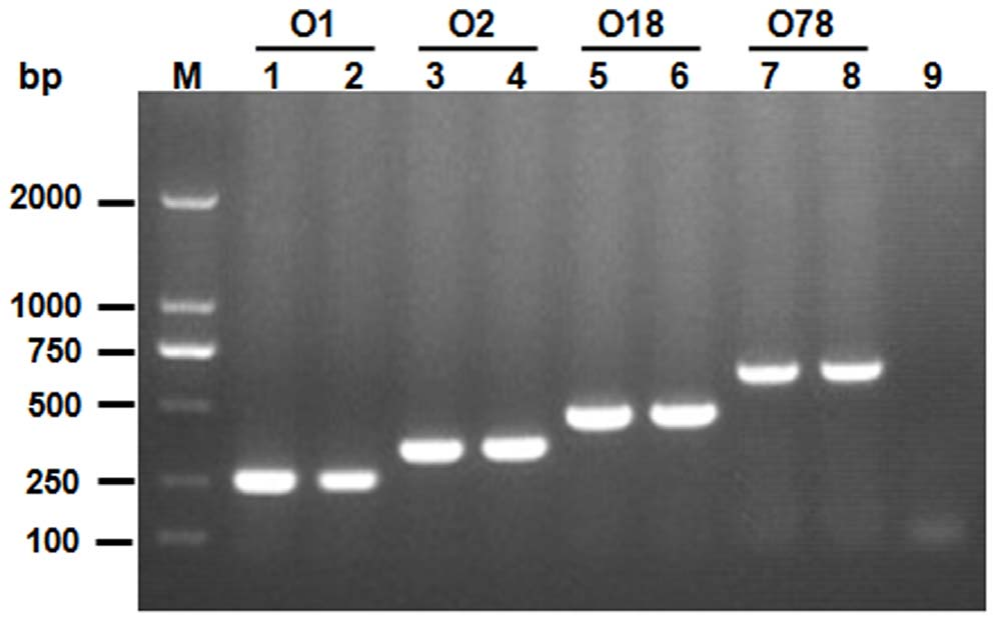
The product profiles of *E. coli* serotypes O1, O2, O18 and O78 strains amplified using the allele-specific PCR. Lane M: DL2000 DNA Marker; O1, O2, O18 and O78 represent PCR products for O1, O2, O18 and O78 strains respectively. Lane 1: APEC O1 strain; Lane 2: APEC strain DE47; Lane 3: APEC strain DE14; Lane 4: APEC strain DE17; Lane 5: APEC strain RS218; Lane 6: APEC strain CE66; Lane 7: APEC strain DE48; Lane 8: APEC strain DE65; Lane 9: Negative control.

The universal forward primer was designed based on the sequence of *gnd* gene. The specific reverse primers were designed based on the glycosyltransferase encoding genes (APEC O1, O2 and O18 strains) and the flippase encoding gene (APEC O78 strain), respectively (Fig. 1). The allele-specific PCR was optimized by adjustment of different parameters, and the resulting optimal condition was described in the Method section. As a result, the serotypes of the reference strains were specifically differentiated by the PCR assays (Fig. 2).

### Determination of the Specificity for the PCR Assays

In order to evaluate the specificity of the primers used in this study, PCR were performed using different bacterial template listed in Table 1. The results showed that the serotype-specific fragments were amplified using respective primers from *E. coli* serotypes O1, O2, O18 and O78 reference strains. No fragment was amplified from other serotypes of *E. coli* reference strains or other species of bacteria tested in the allele-specific PCR assays (data not shown). The PCR products were further confirmed for the specificity by sequencing analysis. The results showed that all primers were specific and compatible in PCR reactions.

The reliability and specificity of the PCR assay was also verified by comparing the sero-typing results of 65 APEC O1, O2, O18 and O78 isolates with serum agglutination assays. As shown in Table 3, 65 isolates were distinctly sero-typed using the PCR assay. However, serum agglutination identified 60 of them, which were completely matched to those of PCR sero-typing. While the other 5 PCR sero-typed isolates, 3 for serotype O78, 1 for serotype O1 and 1 for serotype O2, were shown in a multi-agglutination pattern in the serum agglutination assay. This result further confirmed the specificity of the assay.

**Table 3:** Comparison of PCR and serum agglutination assays for differentiating the serotypes of APEC isolates and clinical infected samples.

Serotypes	APEC isolates (n = 65)		Clinical infected samples (n = 20)	
	PCR	Serum agglutination	PCR	Serum agglutination
O1	9	8	2	2
O2	11	10	3	3
O18	6	6	2	2
O78	39	36	10	9
O1/O2/O18/O78	0	5	0	1

### Assessment of the Sensitivity for the PCR Assays

**Figure 3:**
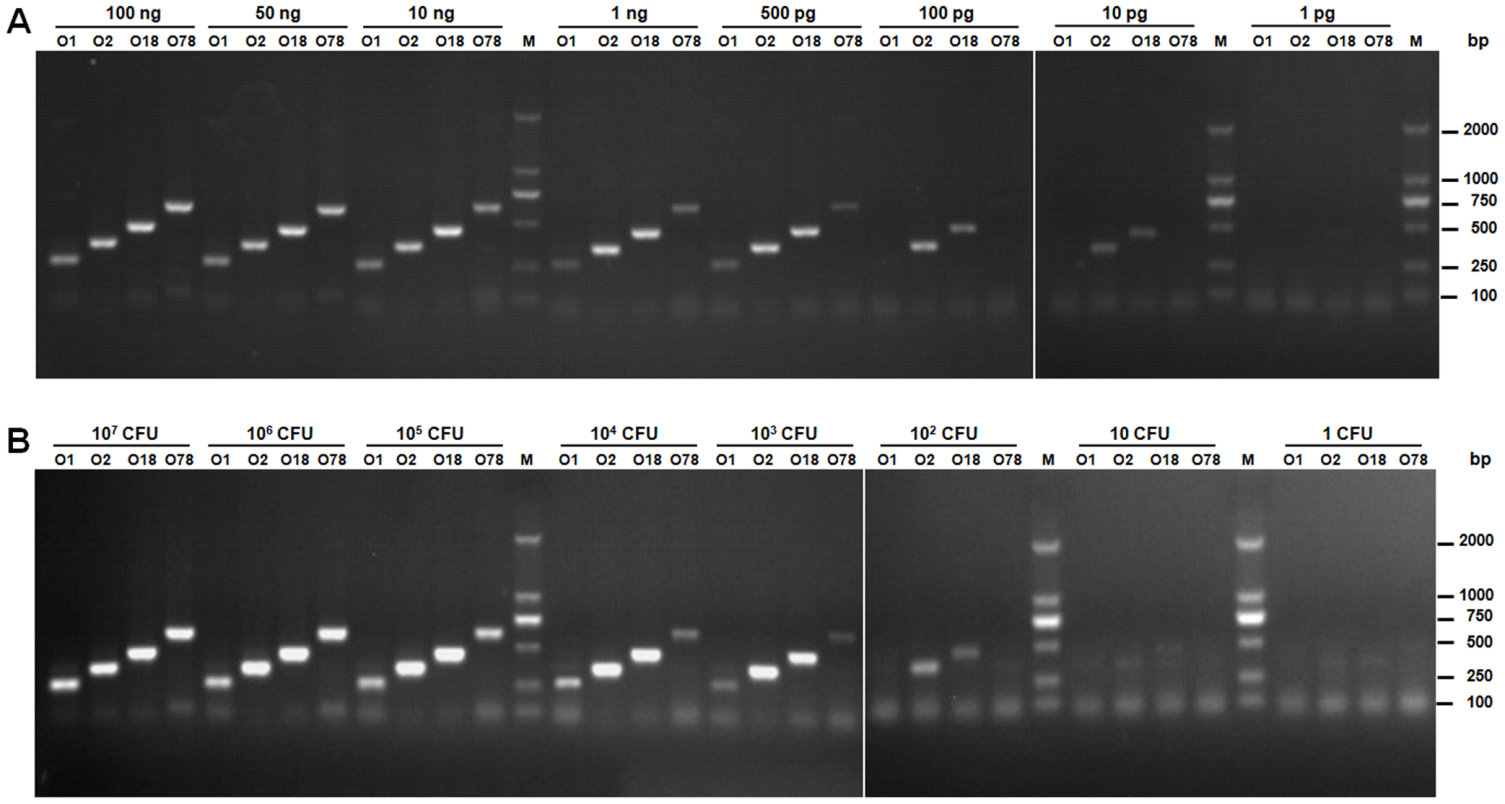
Sensitivity analysis of the allele-specific PCR assay. (**A**) **Sensitivity analysis using the bacterial genomic DNA.** The detection limit was determined as 10 pg of bacterial DNA for *E. coli* serotypes O2 and O18 strains, and 500 pg of bacterial DNA for *E. coli* serotypes O1 and O78 strains, respectively. (**B**) **Sensitivity analysis using the bacterial culture.** The detection limit was determined as 10 CFUs of *E. coli* serotypes O2 and O18 strains, and 1,000 CFUs of *E. coli* serotypes O1 and O78 strains, respectively. Lane M: DL2000 Marker.

The sensitivity of the assay was determined using a series of diluted chromosomal DNA or bacterial culture of *E. coli* serotypes O1, O2, O18 and O78 strains. The results showed that 10 pg DNA or 10 CFUs of O2 and O18 bacteria were sufficient for the amplification of serotype-specific fragments. However, 500 pg DNA or 1,000 CFUs of O1 and O78 bacteria were needed for the amplification of serotype-specific fragments (Fig. 3).

### Sero-typing of the APEC from Experimental or Clinical Infected Samples

The results indicated that 16 isolates from experimentally infected ducks showed corresponding PCR bands at respective size, which was in accordance with bacteriological examination and traditional serum agglutination assay. Four samples from normal control ducks showed no any PCR band. Sero-typing results for 20 clinical samples with colibacillosis showed that 17 of them were serotypes O1, O2, O18 and O78 (Table 3), which was further confirmed by bacterial isolation and regular serum agglutination assay. In order to get more positive samples to verify our allele-specific PCR assay, we collected the samples from APEC positive farms. Therefore, a high positive rate (85%, 17/20) of the predominant serotype strains was obtained, which was similar to that in our previous prevalence studies [7]–[8]. Moreover, one clinical sample, which shows multi-agglutination with O2 and O78 antiserum in a traditional serum agglutination assay, was identified as O78 using this PCR assay. The other 3 clinical samples gave negative results of serotypes O1, O2, O18 and O78 in both PCR and serum agglutination assays, which were belonged to other serotypes (Table 3). The 20 healthy duck samples were negative for serotypes O1, O2, O18 and O78 by bacteriological examination and PCR assay. These results revealed that the developed PCR assay in this study was more sensitive and specific than traditional serum agglutination assay, and it achieved the requirement for the detection of clinical samples.

## Discussion

Colibacillosis is one of the principal causes of morbidity and mortality in poultry worldwide. APEC serotypes O1, O2, O18 and O78 strains are responsible for most of the poultry colibacillosis. Various strains with different serotypes were occasionally found at one APEC outbreak. In addition, the cross-protection among different APEC serotypes is poor [1]–[2], [4]–[7]. Therefore, a rapid and accurate sero-typing method is very important for the APEC control. Serum agglutination assay is a traditional method for APEC sero-typing, however, isolated bacteria and a panel of high quality antisera against different O-antigens are needed for getting the results. Serum agglutination can not simultaneously differentiate APEC serotypes at one test. In addition, occasionally one strain may react with multiple APEC antisera [3]. Thus, an allele-specific PCR assay for sero-typing APEC predominant serotypes was developed in this study.

To design effective primers for the sero-typing, the *rfb* gene cluster and flanking sequence in APEC serotypes O1, O2, O18 and O78 strains were subjected to bioinformatics analysis. The results showed that O-antigen associated genes were highly specific to individual serotypes, suggesting it could be typically used as the target in PCR based typing methods. By taking advantage of these features, a rapid and simple allele-specific PCR assay was developed. The PCR was designed to amplify the specific O-antigen sequences between the *rfb* locus and *gnd* gene at different size. The PCR assay could be used for sero-typing of APEC O1, O2, O18 and O78 strains in bacterial culture and in infected tissue samples, and showed no reaction with other serotypes of *E. coli* reference strains and other species of bacteria, demonstrating a very good specificity of the assay. Moreover, the multi-agglutination isolates could be distinctly sero-typed by this method, suggesting the PCR assay was specific and reliable.

The sensitivity assay of the PCR indicated that a quantity of 10 pg DNA or 10 CFUs bacteria was sufficient for detection of *E. coli* serotypes O2 and O18 strains, and 500 pg DNA or 1,000 CFUs bacteria was needed for detection of *E. coli* serotypes O1 and O78 strains (Fig. 3). Other single PCR system reveals that 1 pg DNA is good for detection of *E. coli* serotypes O15, O174 and O177 [14]–[15]. However, 500 pg DNA are needed to amplify the predominant serotypes of uropathogenic *E. coli* in multiplex PCR assays [21]. The lower sensitivity of multiplex PCR assays may because of possible interference among the primers in the system. The PCR assay was successful for typing of the APEC strains in both experimental and clinical infected tissue samples, suggesting it could be used for clinical and laboratory detection. Moreover, it was able to differentiate the serotypes of the samples with multi-agglutination in the regular bacteriological examination. Thus, this PCR assay was more specific and sensitive than the traditional serum agglutination assay. It achieved the requirements for APEC clinical diagnosis and epidemiology studies with reduced workload and shorted the time.

In summary, an allele-specific PCR assay was developed in this study, which was able to differentiate APEC predominate serotypes of O1, O2, O18 and O78 strains with high specificity and sensitivity. This PCR assay was an efficient and convenient strategy for sero-typing of APEC predominant strains, avoiding the disadvantage of traditional serologic assays. Thus, development of this PCR assay benefits for clinical diagnostics, epidemiology studies, and disease control.
